# Using satellite remote sensing and household survey data to assess human health and nutrition response to environmental change

**DOI:** 10.1007/s11111-013-0201-0

**Published:** 2014-01-04

**Authors:** Molly E. Brown, Kathryn Grace, Gerald Shively, Kiersten B. Johnson, Mark Carroll

**Affiliations:** 1 Biospheric Sciences Laboratory, Code 618, NASA Goddard Space Flight Center, Greenbelt, MD 20771 USA; 2 University of Utah, 260 S. Central Campus Dr., Rm. 270, Salt Lake City, UT 84112 USA; 3 Purdue University, 403 West State St., West Lafayette, IN 47907 USA; 4 Westat, 1600 Research Boulevard, Rockville, MD 20850 USA; 5 Sigma Space Corp, NASA Goddard Space Flight Center (NASA GSFC), Greenbelt, MD 20771 USA; 6 Department of Economics and Resource Management, Norwegian University of Life Sciences, Ås, 1432 Norway

**Keywords:** DHS, NDVI, Environment, Health, Survey, Nutrition

## Abstract

Climate change and degradation of ecosystem services functioning may threaten the ability of current agricultural systems to keep up with demand for adequate and inexpensive food and for clean water, waste disposal and other broader ecosystem services. Human health is likely to be affected by changes occurring across multiple geographic and time scales. Impacts range from increasing transmissibility and the range of vectorborne diseases, such as malaria and yellow fever, to undermining nutrition through deleterious impacts on food production and concomitant increases in food prices. This paper uses case studies to describe methods that make use of satellite remote sensing and Demographic and Health Survey data to better understand individual-level human health and nutrition outcomes. By bringing these diverse datasets together, the connection between environmental change and human health outcomes can be described through new research and analysis.

## Introduction

The past decade has seen a widespread acknowledgment of the profound impact human activities have had on ecosystems and environmental functioning. These impacts have been documented across many ecosystems and continents, and affect nearly all areas of the earth, including those not inhabited by people (Steffen et al. 2011). Ecosystem services, or the benefits provided to humankind by the resources and processes supplied when ecosystems function properly, have long been recognized as critical to human survival. Ecosystems provide human populations with basic needs such as clean drinking water, food, fuel, medicinal plants and buffering from natural disasters. However, rapid changes in climate, land cover and plant and animal community composition can affect the ability of an ecosystem to supply these services; this ability is expected to be further strained in the future (Reid et al. 2005).

The loss of ecosystem services poses a considerable immediate and long-term threat to achieving the millennium development goals of reducing poverty, hunger and disease around the world (Reid et al. 2005). Ecosystem function can change on both short- and long-time scales, with varying levels of resilience to perturbations depending on the geographic and climate conditions where the ecosystem exists. Altered ecosystem functioning may result in a reduction in the ability of human communities to maintain agricultural systems, obtain sufficient or potable water, or access affordable sources of energy without substantial technological and financial investments (Aerts and Honnay 2011). Long-term trends in rainfall and temperature affect how ecosystems function in the future (Dearing et al. 2012). In the short term, ecosystems can be affected by extreme events such as fire, droughts, floods and severe windstorms. These weather events can cause stress on both human and broader ecosystem functioning and can be either narrowly confined or widespread (Yu et al. 2003; Zhou et al. 2003).

Changes in climate and in broader ecosystem functioning may threaten the ability of current agricultural systems to keep pace with growth in population and overall demand for food, fiber and—increasingly—fuel (Dangour et al. 2012). In developing countries, where poor people rely on locally produced food for the majority of their caloric intake, shifts in climate and weather patterns can dramatically reduce agricultural yields (Monfreda et al. 2008) and may have widespread impacts on local economies (Dinar et al. 2008). Reductions in agricultural productivity reduce overall economic activity and cause widespread hardship due to the broad importance of agriculture in these countries. Reductions in food availability and increases in local food prices negatively affect short-term food security in many regions (Brown et al. 2009).

Farming communities are extremely adaptive to changes in climate and natural resource conditions (Crane et al. 2011). As a result of this adaptation, observed changes in environmental conditions, such as those registered by remote sensing, may not necessarily translate into changes in welfare (Mortimore and Turner 2005). Nevertheless, identifying management responses to changes in productivity that allow communities to maintain their health and welfare is a critical step toward understanding how humans can adapt to a changing environment. Other strategies, including seasonal migration and reliance on off-farm earnings, can help to maintain welfare by allowing regions affected by environmental changes to earn incomes or import goods from unaffected regions (Rain 1999). By exploring and measuring the relationships between observed welfare and environmental dynamics, including unexpected relationships, we can help improve policy responses.

Human health is also likely to be affected by climate and ecosystem transformation occurring across multiple geographic and time scales (Kovats and Haines 2005). These impacts range from increasing transmissibility and range of vectorborne diseases, such as malaria and yellow fever, to undermining nutrition through deleterious impacts on food production and concomitant economy-wide increases in food prices.^1^

Little is known about how the specific drivers and interactions of climate and ecosystem change, such as large weather events and other factors, may directly or indirectly affect human health outcomes. This poor understanding primarily reflects a lack of appropriate data with which to simultaneously measure climate and ecosystem change on the one hand, and human health and well-being on the other (Patz et al. 2004). However, recent advances in the collection and use of geospatial data provide an opportunity to explore these linkages by bringing complementary datasets together.

This paper describes methods that make use of satellite remote sensing and population and health survey data, specifically the Demographic and Health Survey (DHS), to better understand drivers of individual-level nutrition outcomes. To illustrate how remotely sensed data can be linked to observations on human health and nutrition, we present four case studies based on recent research from Africa and Asia. Information on how ecosystems are changing and the short-term weather events that are influencing long-term ecosystem functioning can be obtained from satellite remote sensing information (Le Dizès et al. 2003; Nemani et al. 2003; Potter et al. 2003; Zhou et al. 2003). These data provide measures of current environmental status, as well as indicators of changes over time. In terms of human well-being outcomes of interest, the DHS, implemented by ICF International, provides a wide variety of parameters on the health and nutritional status of individuals, including particularly vulnerable populations such as young children and women of child-bearing age. By linking individual, household and community information reported in these surveys to environmental drivers, including remotely sensed information, we can provide new and improved analyses that may permit researchers and policy makers to better understand the impacts of changes in ecosystem functioning on specific human-focused outcomes, as well as the importance of broader weather and climate events for human well-being.

The objective of this paper is to describe a methodological approach to linking satellite remote sensing data and DHS population and health data. We discuss key conceptual and data-related considerations, and then present four case studies of recent analyses that have used these methods to examine research questions at the nexus of environmental/climate change and human health, nutrition and well-being. We conclude by summarizing the benefits and challenges that come with using these two different types of data and suggest avenues for future research.

## Literature review and conceptual framework

Understanding the connections between human health and environmental change requires hypotheses that link landscape and geophysical characteristics with population outcomes and health at relevant spatial and temporal scales (Axinn and Ghimire 2011). Previous work has focused on connecting land use and land cover information derived from remote sensing to household-derived decisions in the few locations where household surveys have been conducted. This broad literature focuses on household decision making and often uses household surveys conducted by the authors themselves (Chowdhury 2007; Fox et al. 2004; Geoghegan et al. 1998). These and other studies have explored connections between household characteristics and land use, but do not focus specifically on health or health outcomes resulting from environmental change or climate variability. Here, we are interested in asking specific questions about health and nutrition outcomes. These include nutritional status, morbidity and mortality. We also explore how researchers might extend the connections we observe locally to entire countries or regions. This paper thus contributes to the literature by providing specific methods that can be used to link geographic information, especially remotely sensed information, to data on health, nutrition and demographic processes in communities and households.

**Fig. 1:**
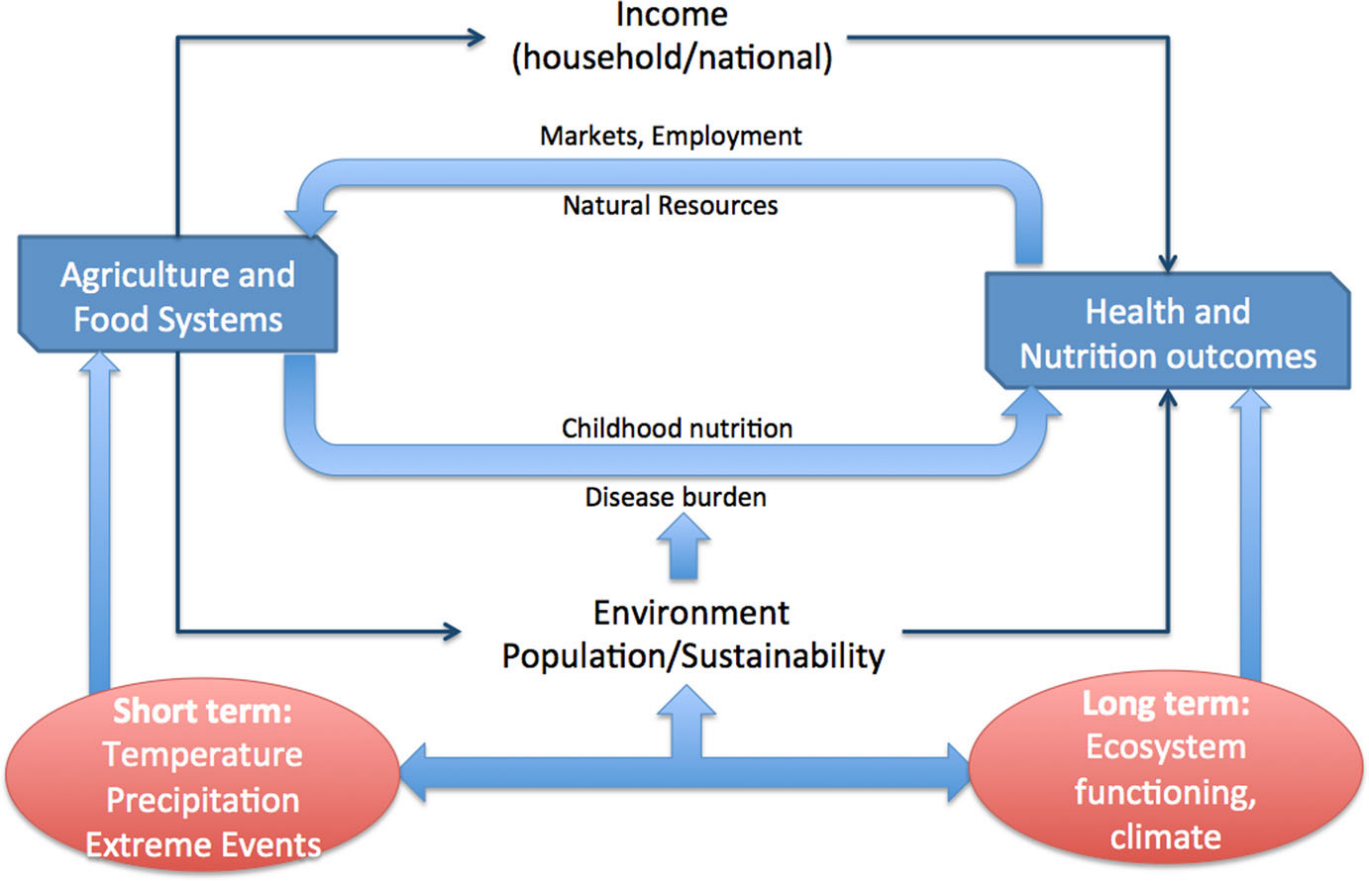
Conceptual framework linking short- and long-term observable parameters to human health and nutrition outcomes

Figure 1 shows short- and long-term interactions between environmental change and health. Food availability and access, mediated by nutrition levels and disease burden, are determinants of health and nutrition outcomes. In the short term, temperature, precipitation and extreme events influence food availability. The impact of agriculturally relevant weather is mediated by such factors as whether an individual or household purchases locally or regionally grown food, or food grown in geographically distant locations and delivered via national or international food markets and systems. Farm households that rely primarily on the food they produce are extremely vulnerable to weather-related production declines. Farm households that produce food for the market are affected by weather through impacts on income. Those that produce both for own consumption and the market are affected via both channels. Social safety nets provided by families, communities, national governments and aid agencies mediate the impact of changing food and income availability on health and nutrition outcomes. In the long run, ecosystem functioning and climate will affect health and nutrition outcomes through changing the resilience and productivity of agricultural and livestock systems, the supply of clean drinking water and safe decomposition of wastes. Interactions between long-term environmental change and short-term agricultural impacts are complex and conditioned by environmental and market characteristics, local population changes and the local environmental impacts of specific household livelihood strategies.

The pathways by which changes in the climate precipitate changes in ecosystems and human health and well-being tend to travel through food production and consumption. We therefore focus our discussion on two food security-related components of this framework: agricultural production and ecosystem services provision.

### Agriculture and health

Agriculture is primarily focused on producing food for human consumption and feed for livestock, but it also generates a range of other intermediate inputs such as cotton, silk, wool, rubber, oil, fuel and medical drugs (Dangour et al. 2012). The agricultural sector is an important source of employment, earnings and foreign exchange, particularly in low-income countries where up to 80 % of the total workforce may be engaged in agriculture (WorldBank 2013). When large-scale reductions in yields occur in any one season, the impact can be felt not only by farmers, but also by wage laborers, truck drivers, market workers and urban consumers. Capturing variation in agricultural production to facilitate understanding of the scope of these impacts requires spatially and temporally explicit information and a clear understanding of how changes in agricultural activity affect population health and nutrition.

### Ecosystem services provision and health

Changes in the broader natural resource base, such as intact forests with a diverse catalog of animal and plant life, clean water and high-quality pastures for livestock, contribute in important ways to human health outcomes. Forests provide a broad range of resources that contribute to improved nutrition outcomes. These include wild animal meat to augment protein and fat in the diets of the rural poor (Golden et al. 2011), diversity of diet and the consumption of vitamin A containing foods (Johnson et al. 2013), and improved clean water sources, resulting in reduced diarrheal disease. Diarrheal diseases are often food- or waterborne and have acute impacts on child nutritional status. Diarrhea is the second leading cause of mortality in children under 5 years of age and is responsible for the deaths of 1.5 million children annually (WHO 2009). Children who are malnourished are mostly at risk of life-threatening diarrhea, and children with diarrhea are more likely to suffer malnutrition as a result (Young and Jaspars 2006).

Another way in which environmental change affects household food security is by altering the supply and quality of heating and cooking fuels. Between 2 and 3 billion people, or roughly 40 % of the world’s population are completely dependent on biomass as their primary fuel for cooking and heating (Openshaw 2011; WHO 2006a). East Africa is particularly dependent on biomass fuels. Rapid land use change is reducing the supply of high-quality biomass in many areas, and particularly in parts of Sub-Saharan Africa. These shortages cause households to shift collection away from forests toward locations such as farms and fields. These sources typically yield much lower per hectare quantities of biomass. As Jagger and Shively (2014) demonstrate for Uganda, such changes in the supply of locally available biomass fuels have implications for household fuel use and the exposure of women and children to harmful gasses and particulate matter associated with the incomplete combustion of low-quality biomass. These changes can have indirect effects on how women and children use their time, the number of meals that are cooked and the types of foods that are prepared. All can affect overall food security as well as health and nutrition outcomes.

Livestock and pastures also are important contributors to nutrition outcomes (Smith et al. 2013). Climate-related degradation of pastures, for example a shift from high-quality, perennial forbs and grasses to inedible plants that cannot support many animals, is a threat to human health outcomes and the livelihoods of pastoral peoples (De Bruijn and van Dijk 2003; Kahsay 2003). Reliance on protein and income-generating resources from livestock mean that severe weather shocks can cause widespread loss of income for the households who own them (Delgado et al. 1999). These same shocks can further degrade already stressed pastoral ecosystems, resulting in the collapse of their ability to support high numbers of animals (Thiam 2003; Wessels et al. 2004).

### Livelihoods as a logical link between environmental change and health

Understanding how individuals and households earn their livelihoods is important for constructing appropriate hypotheses regarding the potential impact of environmental change on human health and nutrition. Additionally, local geography, which affects both the options for production (via climate, soils, etc.) and options for marketing and trade (via roads, proximity to urban centers, etc.), affects household consumption. Items produced and collected by households may be directly consumed, traded/exchanged for other items through barter or sold in the market or through marketing chains. Consumption options are constrained both by availability, i.e., what can be produced or purchased in local markets, and by household purchasing power (FEG 2013). Understanding the range of household livelihood strategies allows a better interpretation of the potential impact of environmental change on a region. As an example, if the population of a region is primarily employed in the manufacturing sector, local changes in rainfall patterns are unlikely to have the same direct impacts on the health and nutrition of the children of wage earners as on the children of subsistence or semi-subsistence farmers.

Livelihood zones, which can be defined operationally and mapped as regions with similar geography, natural and man-made assets, methods of production, and patterns of marketing and trade of goods and services represent an important mediator of the relationship between environmental conditions and human development outcomes.^2^ Similarly, appropriate economic information is necessary when hypothesizing about and measuring relationships between environmental dynamics and health outcomes. If the price of a primary cash crop has declined dramatically in a location where a child resides, or the price of basic foodstuffs has increased, this information ideally should be brought into an analysis of child nutrition outcomes since such changes may have important effects on local food availability and food consumption. Understanding how the broader economic situation interacts with the environment is central to appropriately interpreting the impact of environmental change on human health outcomes (Brown et al. 2009). However, despite the importance of the characteristics and strength of a local or national economy, specific household-level data on economic activity, income and expenditures are typically more relevant for child nutrition outcomes.

## Data

Connecting environmental change to human health impacts requires locally specific, dated and geolocated datasets that can be linked quantitatively. Proxies and indicators of environmental features or changes, and health and population characteristics, can be used to identify broad patterns, trends and potential risks. However, isolating small-scale linkages, e.g., at individual, household and community levels, requires specific, localized information in which specific parameters of interest are provided with accurate latitude–longitude information. In this section, we describe the DHS and the geospatial and remotely sensed data that have been used thus far to examine the relationship between environmental change and human health and nutrition outcomes.

### Demographic and Health Surveys (DHSs)

The DHS are the gold standard source of comparative quantitative data on population, health and nutrition indicators across developing countries (see Table 1 for a comprehensive list of topics covered by the DHS). They are nationally and sub-nationally representative household surveys with large sample sizes that provide detailed information on these topics at a point in time, obtained by interviewing eligible respondents in selected households. Data collection typically focuses on women aged 15–49, men aged 15–59 and children below 5 years of age. The datasets also include information on household and other socioeconomic characteristics.

**Table 1:** Standard population and health topics covered in the Demographic and Health Surveys

Questionnaire topics	Reported indicators
Anemia	Prevalence of anemia, iron supplementation
Child health	Vaccinations, childhood illness, newborn care
Domestic violence	Prevalence of domestic violence and consequences of violence
Education	Literacy, attendance, highest level achieved
Environmental health	Water, sanitation, cooking fuel
Family planning	knowledge and use of contraceptives, unmet need for family planning
Female genital mutilation	Prevalence of and attitudes about female genital mutilation
Fertility and fertility preference	Total fertility rate, desired family size, marriage, sexual activity
HIV/AIDS knowledge, attitudes and behavior	Knowledge of HIV prevention, misconceptions, stigma, higher-risk sexual behavior, previous HIV testing
HIV serostatus	Prevalence of HIV by demographic and behavioral characteristics
Household and respondent characteristics	Electricity, housing quality, possessions, education and school attendance, age, sex and employment
Infant and child mortality	Infant, child and under five mortality rates
Malaria	Ownership and use of mosquito nets, prevalence and treatment of fever, indoor residual spraying for mosquitoes, rapid diagnostic testing and parasitaemia
Maternal health	Antenatal, delivery and postnatal care
Maternal mortality	Maternal mortality ratio
Nutrition	Child feeding practices, vitamin supplementation, anthropometry and salt iodization
Tobacco use	Tobacco use, exposure to second-hand smoke
Wealth index	Asset-based relative wealth index
Women’s empowerment	Gender attitudes, women’s decision making power
Other modules	Fistula, health expenditures

Demographic and Health Survey (DHS) data are collected from probability samples selected using a stratified two-stage cluster design. In the first stage, enumeration areas (EAs) are drawn generally from the most recent census files. In the second stage, a sample of 25–35 households is drawn from an updated list of households within each enumeration area. This group of households constitutes the sampling *cluster*.

The DHS does not generally follow specific households or children over time, or provide historical information on household variables, even though many of the nutrition-relevant variables of interest in the DHS surveys [such as low height for age (HAZ), and low weight for height (WHZ)] may be shaped by events that occurred months or years prior to child measurement. This adds additional value to efforts to link remotely sensed environmental information to nutrition outcomes. Historical remotely sensed data provides insight into broader resource availability. If current conditions differ from historical, this gives an indication of a loss of ecosystem services due to the human impact on the region or potentially to climate change.

**Fig. 2:**
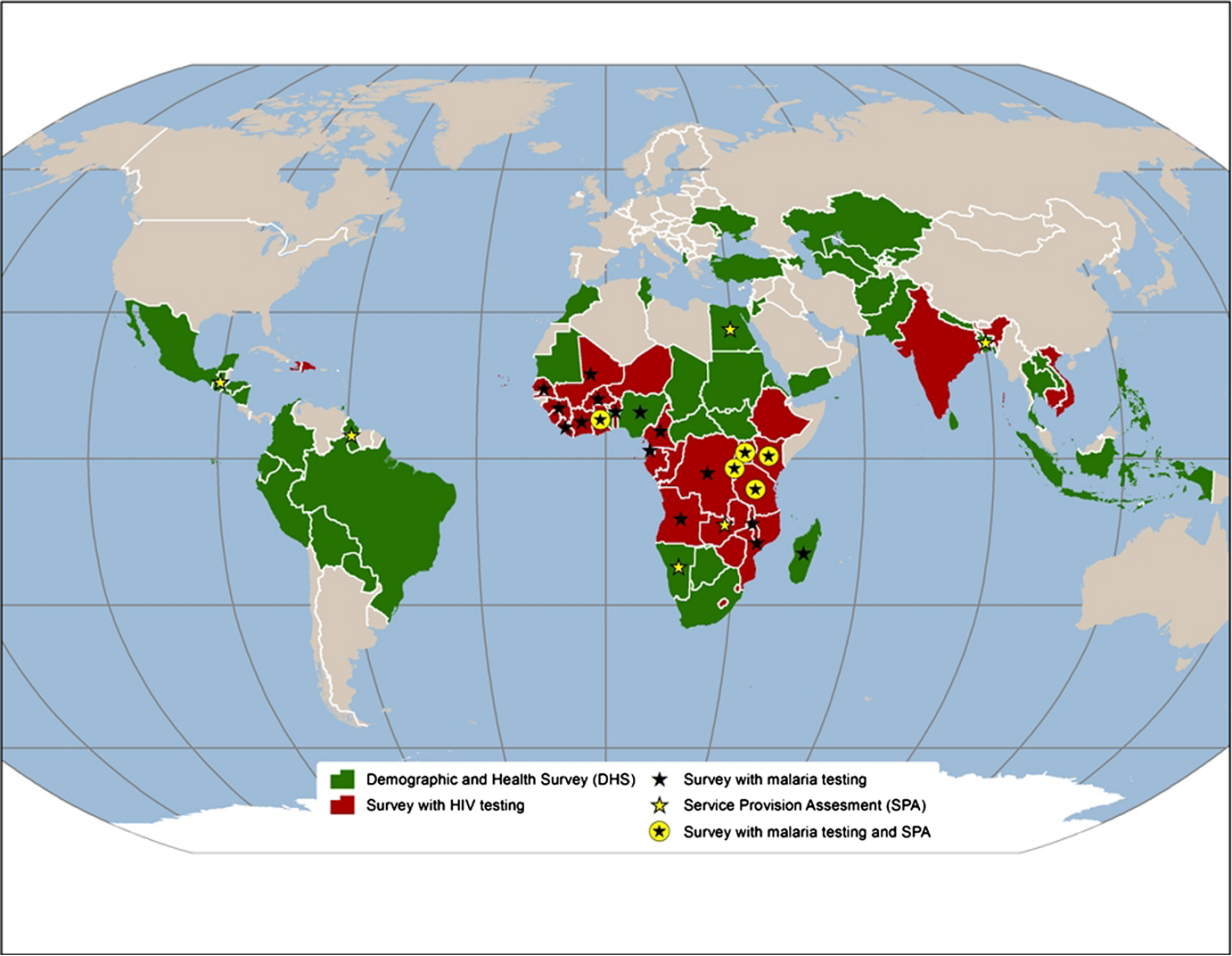
Map of the coverage of the Demographic and Health Surveys project

Demographic and Health Survey (DHS) observations are weighted to be representative at the national level, according to urban/rural residence and at the provincial level (departments, states). More than 300 DHS surveys have been implemented in over 90 countries since the inception of the United States Agency for International Development (USAID)-funded project in 1984 (see Fig. 2).^3^

Since the mid-1990s, DHS has collected geographic information in most surveyed countries at the level of the cluster. The latitude and longitude of each household cluster allows the connection of environmental information to the survey results. During DHS fieldwork activities, the global position system (GPS) coordinates for the approximate center of the populated area surveyed (cluster centroid) are collected using handheld GPS units. During data processing, GPS coordinates are displaced to ensure that respondent confidentiality is maintained. The displacement is randomly applied so that rural points contain a minimum of 0 and a maximum of 5 km of positional error. Urban points contain a minimum of 0 and a maximum of 2 km of error. A further 1 % of the rural sample points are offset a minimum of 0 and a maximum of 10 km. This random shift eliminates the possibility of calculating exact distances from the cluster to other locations of interest and requires that a buffer of some type is used when linking the DHS and landscape/geophysical data.

### Geographic and remote sensing information

Biophysical parameters observed with satellite remote sensing can be used to help verify and test the potential drivers that influence observable human health and demographic outcomes. Remotely sensed data are direct measures of the light or electromagnetic radiation reflected from objects on the earth or from the earth itself. These measures occur on spatial scales of sub-1 m to tens of kilometers and in temporal resolution from hourly to annual and decadal measures. The direct measures of reflected energy are interpreted to produce categorical representations of the earth surface that represent the type of surface (land or water; soil or vegetation; etc.) or some phenomena (e.g., rainfall, fire) occurring at the surface. These categorical representations relate to specific biophysical parameters. For example, the normalized difference vegetation index (NDVI) is a ratio of the light reflected in the red portion (RED) of the electromagnetic spectrum versus the light reflected in the near-infrared (NIR) (Tucker 1979). The equation for NDVI is:

$$ {\text{NDVI }} = \, \left( {{\text{NIR}} - {\text{RED}}} \right)/\left( {{\text{NIR}} + {\text{RED}}} \right). $$

**Fig. 3:**
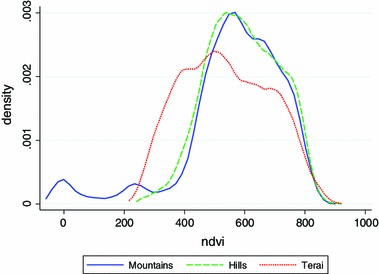
Distribution of average NDVI values (×1000) for the month of September for the three major agroecological zones in Nepal, 2000–2011

The index provides a measure of the greenness of the ground cover, which can be used as a proxy for the productivity and yield of a cereal crop (Brown and de Beurs 2008; Hicke et al. 2004; Prince et al. 2001; Reynolds et al. 2000; Shuttleworth and Wallace 2009). In this paper, we use vegetation data from the moderate resolution imaging spectroradiometer (MODIS), whose data start in 2000, and the advanced very high resolution radiometer (AVHRR) whose data start in 1981. One can analyze many consecutive measurements of NDVI over weeks, months or years to gain an understanding of how vegetation is performing in a region over time. Representative NDVI data for Nepal are graphed in Fig. 3.

Table 2 lists remote sensing datasets that are currently available and which can be used to assess various impacts of environmental change and dynamics. These datasets can be used to assess short- and long-term changes in ecosystem functioning, including the effect of weather extremes, land cover change and species shifts due to ecosystem changes.

**Table 2:** Available remotely sensed and modeled data that provide indicators that could be linked to human health and nutrition

Sensor	Measurements	Time	Resolution
*Observed data*			
AVHRR	Vegetation density/greenness, land surface temperature	30+ years Daily repeat 1981–2013	1–8 km
MODIS	Forest cover (loss/gain), land cover, vegetation density/greenness, land surface temp and evapotranspiration	13+ years Daily 2000–2013	250 m–1 km
Landsat	Land cover, cover change (forest loss/gain, desertification)	40+ years 16 Day repeat 1970–2013	30–90 m
VIIRS	Vegetation density/greenness, land surface temp	1+ year Daily 2012–2013	375–750 m

Dataset name	Measurements		Resolution
*Modeled data*			
MERRA	Precipitation, temperature, wind, evapotranspiration and pressure	3 Hourly data	1/2°
NASA land data assimilation	Precipitation, temperature, wind, evapotranspiration and pressure	Daily data	1/4° and 1°
IPCC	Predicted climate variables such as temperature, precipitation, etc.	Monthly data	3°

For a broader regional view, remotely sensed data can be combined to produce land cover type (forest, agriculture, desert, etc.). These types of products are usually produced infrequently (annually to decadally) and give an overview of the region and the functioning of its ecosystems. The land cover products can be linked to the other remotely sensed parameters to produce measures of the “state” of a land cover type. For example, one could extract all of the NDVI values over a certain time period for areas that are categorized as “forest.” Analyzing this dataset could provide indicators of forest health or variability in forest health.

Interpreted remotely sensed data products come in two general forms. Discrete variables provide a specific numeric value in the data file that refers to a specific condition on the ground (e.g., 1 = forest, 2 = water, etc.). Continuous variables are defined over a range of values (e.g., 0–100 % forest cover), and values reported in the data files refer to a gradation along that continuum. The different applications of these data types will be discussed in detail in the following sections. Remotely sensed data have historically been used to describe the physical condition of a place at a specific point in time or over a specific interval. They have also been used to establish baseline conditions for models of environmental factors such as hydrology or climate. The data record of biophysical parameters is growing and provides an opportunity to exploit the increasing length of this data record to provide a measure of the ecosystem services available in a given region and how those services may be changing through time. Linking this information to information available in the DHS on specific individuals provides an opportunity to uncover associations between environmental conditions and human health and nutrition outcomes.

### Other data sources

As mentioned above, specific household-level data are important to understanding the nutrition outcomes of children. Such data may include the range of economic activities undertaken by a household, its choice of crops, the overall level of agricultural production, or income and expenditures. All are typically relevant for understanding child nutrition outcomes. However, the DHS surveys do not include very much detail on many of the relevant variables in this category.

Such data are often available through data collection efforts of national governments or through The World Bank’s Living Standards Measurement Surveys. Increasingly, such data are being made available with geolocator information, which introduces opportunities for connecting them, albeit approximately, to DHS and remotely sensed data. In addition, an increasing number of household panel surveys are being made available. These surveys, which follow individuals or households over time at regular intervals, open up the opportunity to measure the changes and volatility in economic variables, which may be especially relevant to child nutrition.

### Discrete and continuous remote sensing variables

Discrete variables represent the earth’s surface with values that can be directly mapped to a specific feature or type of feature. In most cases, these are land cover classifications but can also be used for many binary representations such as presence or absence of a feature, change or phenomena (such as fire). Land cover classification of remotely sensed data has been performed since the first earth observing satellites started collecting data. There are many different schemes tailored to specific communities but all share a common heritage and utilize various thresholds of the physical measurements to create boundaries between different geographic regions. The regions can be forest patches, roads/impervious surfaces, agriculture fields, water, savanna, etc. The number and type of categories are defined by the purpose of the map, but there has been effort to centralize this into a common method through the IGBP (Townshend 1992) and the Land Cover Classification System (LCCS) a hierarchical system that allows a user to pick how many levels of distinction she wishes to represent, using a common set of terms to describe the classes (Bartholome and Belward 2004; DiGregorio and Jansen 2000).

Most of the products generated from remotely sensed data are continuous variables from the actual direct measurements of radiance and reflectance, to derived indices such as NDVI or leaf area index (LAI), and finally to land cover representations such as the vegetation continuous fields (VCFs) from moderate resolution imaging spectroradiometer (MODIS). Continuous variables work by defining a range of values that will define a phenomenon (Carroll et al. 2011). The VCF product is percent cover so the valid range is 0–100 %. Where discrete values provide definitive boundaries between features, the continuous variables allow for gradations between features since there are greater numbers of values to assign.

### The importance of resolution, scale and time in remote sensing

All remotely sensed data are characterized by three type of resolution: “spatial,” “spectral” and “temporal.” These dimensions define the qualities of the data itself. Spectral resolution describes the wavelengths of the electromagnetic spectrum that the sensor measured. This can range from sub-visible through thermal, microwave and radar. Spatial resolution describes the minimum unit on the ground that was observed by the sensor. 30 m spatial resolution indicates that each measurement represents an area on the ground that is 30 m on a side or 30 m^2^. Coarser spatial resolution (e.g., 250 m, 8,000 m) typically indicates that the measurements represent more than one type of feature on the ground. For example, data points with course spatial resolution typically encompass a landscape mosaic that might include trees, grass, agricultural fields, waterways and roads. Temporal resolution describes how much time a data point represents and when it was collected. It could consist of a single image from 1 day, or it might represent the average of a set of images collected over many days. In most cases, there is a trade-off between spatial and temporal resolution: sensors with coarser spatial resolution typically see the same location on earth with greater temporal frequency.

## Methods for geographically linking environmental change to health outcomes

Using satellite data to observe environmental threats requires a clear theoretical connection between the biophysical variable and the environmental threat to human health. The following provides a survey of how remote sensing can be used to estimate environmental changes that are relevant to health impacts.

### Linking household clusters to environmental parameters

Linking the cluster centroid to remote sensing information requires a statistical approach that uses a quantitative assessment of the landscape. The random displacement of the DHS data prevents direct measures of the exact locations of households or children, but does not prevent one from identifying more general relationships between overall conditions, availability of resources and outcomes. In this case, it is useful to rely on the first law of geography, which states that “everything is related to everything else, but near things are more related than distant things” (Tobler 1970).

In a recently completed study, the MODIS VCF percent tree cover product (Hansen et al. 2002, 2003, 2005) was used to characterize the density of forest cover for DHS sampling clusters in Malawi. The native spatial resolution of the VCF is 250 m, which means that every value is relative to a spot on the ground that is 250 m^2^. Interannual variability in the remotely sensed data can result in fluctuations in the output values in the VCF (and all annual remote sensing products). To account for this, the study collapsed VCF data and used an average value to generate a single product to link with the DHS observations. Johnson et al. (2013) computed an average across 3 years of data (2008–2010) to observe close to the sample date of the DHS to represent the forest cover for the relevant time period represented in the DHS. This method is potentially more robust to short-term fluctuations in remotely sensed data than relying upon a single year of observed data (Johnson et al. 2013).

To account for the locational displacement of the DHS observational units, the VCF and other remotely sensed data are typically aggregated to a commensurate resolution by exact averaging. For example, in Johnson et al. (2013), each observation within a 5-km grid cell was summed and divided by the total number of observations within the 5-km grid, excluding any observations of water within the 5-km grid cell. The DHS points were then overlaid with the percent tree cover. The value of percent tree cover that the point fell within was selected to represent the cover type for this point. This method provides a measure of the conditions in the general area of the sample survey location.

### Statistical models to link health outcomes to environmental change

The DHS surveys focus attention on measurements of child health and nutritional status. These are generally characterized by three anthropometric measures: height-for-age, weight-for-height and weight-for-age. When evaluating populations of children, *Z* scores are used for anthropometric measurements. Underlying growth measures to compute *Z* scores are collected for all eligible children and expressed in terms of the dispersion of the child health indicator as standard deviations around a reference population mean. The *Z* score is calculated as:

$$ z_{i} = \frac{{x_{i} - \bar{x}}}{{\sigma_{x} }} $$

where *x*_*i*_ is the individual observation and $\bar{x}$ and *σ*_*x*_ are the median and the standard deviation of the reference population. *Z* scores are linear and independent of sex. Normal growth patterns of children under the age of five who are well-nourished exhibit similar heights and weights, despite geographic, ethnic and cultural differences (Habicht et al. 1974). Any departure from this distribution of optimal growth, therefore, can be attributed to socioeconomic and environmental factors. *Z* scores are typically calculated using WHO’s current child growth standards reference population median and standard deviation. A child’s height-for-age *Z* score (HAZ) reflects impacts of health and/or nutritional conditions on growth and development during gestation and exogenous factors that affect the child after birth. A low HAZ value (i.e., *stunting*) is associated with a number of long-term causal factors such as insufficient food intake and an unhealthy physical environment. A low HAZ value is generally accepted as a strong indicator of long-term nutritional deficiency and/or repeated illness. A child’s weight-for-height *Z* score (WHZ) is a shorter-term measure of nutritional status that is sensitive to more recent and severe events. Substantial weight loss is usually a consequence of recent disease or illness and/or lack of food. Finally, a child’s weight-for-age *Z* score (WAZ) is a combination of height-for-age and weight-for-height, and measures both chronic and acute malnutrition (Puffer and Serrano 1973). Children are considered *stunted*, *wasted* or *underweight*, respectively, if their height-for-age, weight-for-height and weight-for-age *Z* scores are below −2.0. If a *Z* score falls below −3.0, a child is considered severely stunted, wasted or underweight (WHO 2006b).^4^

Because the DHS data are collected with attention to community clusters, it is not always appropriate to treat individuals within the same community as independent in statistical analyses. Individuals may share many of the same traits—both observed and unobserved characteristics—and may tend to have similar corresponding environmental data because locations close to each other have more similar environmental parameters than places geographically distant. To account for the lack of independence among these observations as well as to incorporate the fact that rainfall, agriculture, temperature and other environmental variables may impact individuals through their impact on the community, multi-level models are ideal.^5^ A multi-level or hierarchical modeling strategy allows the analyst to account for variance between individuals as well as between community clusters. Multi-level models can be structured to accommodate a categorical variable, for example stunted versus not stunted or low birth weight versus normal birth weight. Categorical variables are often used to characterize health and population outcomes.

Environmental region, ecological zone or community cluster can be treated as a random effect in a regression model, while other variables measured at the level of the individual or the household can be treated as fixed effects. This strategy can be used to account for variations in economic parameters such as inflation rate and potential unmeasured variation in factors related to pricing or politics, which may have relevance on a larger spatial scale like the livelihood zone or a political jurisdiction. Additionally, environmental outcomes like drought or drought risk can be used as a random effect. Because the context—environmental or economic—is often conceived of at a larger spatial scale than the household, multi-level models provide an excellent empirical strategy for incorporating variation due to differences within and across different groups of interest.

### Linking data at appropriate points in time

A weight-for-height *Z* score is an inherently short-term indicator of acute food shortage or compromised health. In contrast, a height-for-age *Z* score is a longer-term indicator of chronic food shortage or compromised health. Because these nutrition indicators may reflect environmental conditions that prevailed during different time periods in the child’s history, it may prove advantageous during analysis to explicitly link remotely sensed data to nutrition-sensitive periods in the child’s life. Furthermore, if environmental outcomes are hypothesized to reflect agronomic conditions, such as growing conditions in a local area, it may be necessary to account for the crop calendar and relevant growing periods for the most commonly grown crops in the vicinity of a household.

**Fig. 4:**
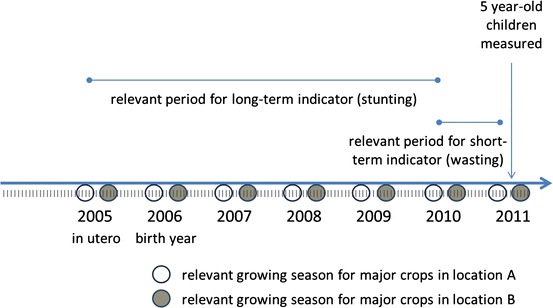
Example nutritional timeline for two identically aged children living in two different agroecological zones

Figure 4 provides a diagram that highlights these issues via a timeline, where arrows indicate relevant nutritionally sensitive periods in the life of a representative child. As an example, consider two children with identical birth dates who are measured in 2011 at the age of 5 years. For these children, the relevant period for a short-term nutritional indicator such as WHZ (wasting) might be the month immediately prior to anthropometric measurement. In contrast, for the same children, the relevant period for a long-term nutritional indicator such as HAZ (stunting) might extend well beyond the most recent 12-month period and could include the first year of life or even the period immediately prior to birth, when the mother’s gestational health is paramount to the child’s development. By extension, the nutritional outcomes of children of different ages residing in the same location may be sensitive to remotely sensed data with different time stamps. Moreover, children of similar ages, but residing in locations where the crops most important to the household differ, may have nutritional profiles that are sensitive to slightly different periods in the calendar, due to differences in growing periods. Such differences for the children in this simple example are indicated by the circles in Fig. 4, which illustrate different, location-specific growing periods critical to food production and nutritional intake.

## Results: case studies

### Moisture conditions and mortality in West Africa

Connecting NDVI with DHS information can help policy makers understand the consequences of a variable environment on childhood mortality and nutrition. Because the DHS data are based on independent observations of individual children, the impact of environmental dynamics can be assessed. A study from West Africa linked NDVI in the growing season prior to the reference child’s date of birth to child nutritional status (stunting and wasting) and child survival in 4 countries: Benin, Burkina Faso, Guinea and Mali. The association between environmental conditions (as proxied by vegetative growth) and child survival and nutritional outcomes was assessed using multivariate methods. Nutritional status and survival data, as well as other individual-level characteristics, were drawn from the Demographic and Health Surveys (2001 in the case of Benin, 2003 for Burkina Faso, 2005 for Guinea and 2001 and 2006 for Mali). We used different years and different countries to compare the impact of growing conditions across multiple countries and agro-ecosystems (Johnson and Brown 2010).

The methods used were a one-way ANOVA with a bivariate analysis of the relationship between NDVI and the outcome variables of interest. For our multivariate analysis, a logistic regression was used to examine the correlation between our independent variable of interest (NDVI) and our selected outcome variables, while controlling for confounding factors for which data were available. Results were expressed in odds ratios that indicate the associations between the independent and dependent variables without attributing causality.

The study is unique because of its multi-country design and its use of satellite data to assess drought. West Africa is expected to be particularly hard-hit by the effects of climate change due to the region’s dependence on rainfed agriculture as a primary livelihood and poor regional capacity to adapt to changing conditions. By using observations of children’s nutrition and health parameters of stunting, wasting and mortality, the study aimed to understand the broad implications of environmental change that persist even in the face of enormous investment by local, national and international aid and emergency response programs.

### Ecosystem services and health outcomes: the example of Malawi forests

Following on the definition provided above, one way to model the factors associated with child malnutrition is to study the proportion of children below the WHO *Z* score cutoff for malnutrition. A study in Malawi (Johnson et al. 2013) used DHS data to assess the impact of deforestation on human health. The VCF product was used to estimate forest cover and decadal change in forest cover for the sampled DHS cluster (Hansen et al. 2003). We used binomial logistic regression to predict the odds of selected outcomes, holding constant potentially confounding variables. We focused on associations between biodiversity-related predictor variables and selected dichotomous outcomes (diverse diet = 1, else = 0; consumption of vitamin A-rich foods = 1, else = 0; experience of diarrhea in the past 2 weeks = 1, else = 0). We found that deforestation was associated with poor dietary diversity (few vitamin A rich fruits or vegetables) and increased incidence of diarrhea during the previous 2 weeks before the DHS instrument was administered. Because deforestation was defined as a change in percent tree cover over a decade (2000–2010), the long-term change in ecosystem services provided by these trees could be evaluated. By using the satellite data to evaluate deforestation in a comprehensive and spatially explicit manner, the study revealed systematic relationships that otherwise would be difficult to observe.

### Food prices, agricultural productivity and children’s birth weight in Kenya

In a Kenyan study, Grace et al. (2013) examined the birth weights of infants, a health outcome that reflects a woman’s nutritional status during pregnancy. The retrospective nature of the DHS allowed the researchers to examine birth weights, as recalled by the mother, of her most recently born children. Birth weights were classified as healthy or low birth weight using the WHO cutoff value of 2,500 g. Community NDVI and local maize prices were associated with each birth for each of the 12 months preceding each birth—the preconception and pregnancy periods. NDVI served as a measure of food production (food availability) in the community, while maize prices, provided by the Famine Early Warning Systems Network and USAID, served as indicators of food availability. Maize price data were only available for a small selection of major markets in Kenya. Because the livelihood zone data provided by FEWS NET reflects the dominant strategy used to produce food and household income in an area and are constructed with attention to markets, these zones were used to group community clusters. In other words, all communities that were located within a specific livelihood zone were assumed to be subject to the same price patterns represented by those of the major markets in the zone. The researchers assumed that an increase in maize price in one livelihood zone would reduce access to one of the most important food items relied on by Kenya’s poor for all residents in that livelihood zone. This price increase would indicate a general reduction in food access of the populations most likely to face food insecurity (Grace et al. 2013).

Multi-level regression analysis was used with livelihood zone as the random effect (or nesting variable). The results ultimately revealed that prices have an impact on birth weight outcomes but only as they interact with NDVI. When NDVI, a measure of local food production, is high, then there are fewer cases of low birth weight. Furthermore, the likelihood of low birth weight is especially reduced when NDVI is high and when prices are low. Prices on their own, however, had little impact on birth weight outcomes in the analysis (Grace et al. 2013).

### Agricultural productivity and child stunting in the agro-ecosystems of Nepal

Remotely sensed data were incorporated in a study of agriculture and child nutrition outcomes in Nepal (Sununtnasuk 2013). In Nepal, sources of nutrition are often determined by local agricultural conditions because poor infrastructure, harsh terrain and high transportation costs frustrate efforts to redistribute food from food-surplus to food-deficit areas. NDVI measures were matched to data from the 2011 Nepal DHS. The combined data were used in a series of Probit regressions to evaluate whether interannual variability in weather and its impact on food production was correlated with a child’s probability of being stunted or wasted (Sununtnasuk 2013). The hypothesis motivating this analysis was that NDVI values might help to predict vegetation patterns which could translate to food availability and, ultimately, consumption patterns of household members (Sununtnasuk 2013).

Because losses in height-for-age relative to the global reference group are rarely fully recovered after the second year, leaving the effects of stunting largely irreversible, only children above the age of two were considered for analysis. The nationally representative sample included 1,412 children who were above the age of 24 months at the time of the DHS survey. The binary response variable was recorded as one if the observed child was stunted (HAZ < −2.0) and zero if the child was not stunted (HAZ > −2.0). Transforming the continuous *Z* scores into a binary indicator resulted in a loss of information; however, the objective of the analysis was to examine the relationship between NDVI measures in determining child stunting, and not the partial effects of these on *Z* score outcomes. NDVI values were measured as anomalies, i.e., differences between monthly NDVI values (constructed from daily observations) and the long-term average NDVI for that month computed over a longer period (in this case July 2002 to May 2012). To more carefully connect NDVI values to critical period in the child’s development, they were matched to children according to local, crop-specific agricultural calendars, along the lines illustrated in Fig. 4. In other words, NDVI anomalies were connected to children on the basis of those months that were likely to have been most important for crops grown in the cluster in which the household resided. Several different time periods were considered in the regression analysis. Results from regressions that included a full set of control variables measured at the child, household and community level indicated that positive NDVI anomalies for harvest months during the time a child was in utero were associated with modest, but statistically significant reductions in the probability of stunting (Sununtnasuk 2013).

## Conclusions and recommendations

Changing climate and ecosystem transformations often occurs simultaneously with many other societal, economic and geopolitical changes in the communities residing in these ecosystems. Discerning the impact of environmental change on human health and nutrition outcomes will require separating the effects of changing environment from the many other factors that can affect these outcomes. Recommendations for improved data for analysis of impact of environmental change on human health and nutrition outcomes include:

- Surveys that incorporate observations of nutrition outcomes using standard approaches to better understand the impact of environmental change and extreme events;
- Household surveys that have a protocol for allowing undisplaced GIS data to be linked to external datasets, including remote sensing products, without loss of confidentiality of survey respondents, and without loss of spatial accuracy; Use of satellite remote sensing that permits spatially explicit and temporally frequent observations of environmental change and functioning together with nutrition information; and
- Exploration of hypotheses of environmental impact on nutrition outcomes for a variety of regions and cultures.

Important differences in nutritional status have been documented among villages within the same locality. For this reason, remote sensing data, with their high levels of geographic specificity, could improve targeting of development and intervention programs focused on reducing rural poverty. Differences in water supply may have accounted for local village-wise differences in children’s anthropometric status in northern Nigeria (Tomkins et al. 1978). Lack of clean drinking water, in addition to measles vaccination rates, helped to explain statistically significant differences among villages in mortality rates during the Darfur famine in 1984 and 1985 (De Waal 1989). These examples show the potential of using spatially explicit information to identify environmentally driven threats to human welfare.

Having appropriate mapping of livelihood zones, perhaps in addition to or even instead of the frequently used political boundaries, will enable the clustering of households into groups where dependence on the environment is more homogenous. Small area estimation techniques can be used to improve the estimation of critical human health outcomes in regions where the sample size from a household survey may be too small to accurately estimate the impact (Fay and Herriot 1979). Using spatially explicit satellite remote sensing of important parameters may allow researchers to create estimators of health outcomes that have increased accuracy and spatial specificity.

Differential impacts of annual seasonal hunger also have been documented anthropometrically on relatively fine spatial scales. Seasonal nutritional impacts on children differed significantly between two neighboring villages in Tanzania (Wandel and Holmboe-Ottesen 1992). Seasonality in food availability (Brown and de Beurs 2008; Husak et al. 2013) and in food prices and thus food access (Becquey et al. 2012; Devereux 2012; Sagn 1998) can have important impacts on food security outcomes, which vary depending on where an individual resides (Hillbruner and Egan 2008). Remote sensing science can provide links between season nutritional stress and land cover, and its differential response to climate variability at a resolution that can resolve differences in fields, communities and regions.

Many factors contribute to child nutrition outcomes. Some are more easily observed and measured than others. Going forward, researchers will need to find ways to incorporate variables such as food prices, food quality, time allocation and measures of isolation and risk. Expanding the set of explanatory variables including in these analyses will be critical to understanding the impact of an environmental stressor in a specific location. In addition, how vulnerable a community or household might be to a particular environmental extreme captured with satellite data may depend on the resilience of households and communities and their ability to draw resources from elsewhere. Adding these additional and nuanced features to datasets will be challenging, since they are often disparate in the spatial and temporal resolutions.

The pace at which environmental transformation is occurring is rapid. For this reason, demonstrating clear, documentable connections between observed change and human health and nutrition outcomes will enable focused policies and humanitarian investments that appropriately link the health, nutrition, food security and environmental sectors. Without acknowledgment and exploration of these linkages, each sector alone is unlikely to make substantive progress on their goals. This has long been recognized, but little progress has been made in integrating metrics and programs seeking to improve environmental performance or health outcomes.

Interdisciplinary work requires interdisciplinary teams, and without substantial efforts to integrate expertise across multiple disciplines, this work will not be possible. Effectively analyzing the impact of environmental change requires joint quantitative analysis of human health and nutrition, the environment and economic performance. This requires expertise across all sectors at the beginning of a study, and approaches that incorporate and ground-truth the most precise data collected at appropriate spatial and temporal scales. Creatively combining and validating existing data, and improving and coordinating the collection of future data, are research imperatives.

## Footnotes

1 Ecologically coupled diseases and economy-wide impacts are important, but beyond the scope of analysis and discussion in this paper, which focuses on local changes.

2 The USAID Famine Early Warning Systems Network and the UN World food program have funded livelihood zone mapping for the most food-insecure regions. These maps are produced using expert elicitation and knowledge of a region; production involves six steps, beginning with a general disaggregating or zoning of geographic areas according to similar livelihood patterns and access to markets. Step two involves classifying households into common wealth groups and asset ownership. Once the wealth groups are determined, focus group interviews are conducted with members of households in each group to estimate food access, income and expenditures FEWSNET (2009).

3 More information about the DHS surveys can be found at http://measuredhs.com.

4 HAZ and WHZ are frequently used in combination as dependent variables in linear regressions. If these outcome variables are studied simultaneously with different sets of regressors, and if disturbance terms in the two regressions are correlated (as is likely), gains in efficiency can be obtained by estimating the pair of regressions as a system, using seemingly unrelated regression (SUR) models Greene (2008). Correlated errors could arise, for example, if measurement errors in Z-scores arise from mismeasurement of child age, which influences both variables.

5 These types of models are referred to by several different names—random effects models, regressions with varying intercepts, hierarchical models, etc. (see Gelman and Hill 2006 for an in-depth discussion of these methods) Gelman and Hill (2006).
